# Liposomes Prevent In Vitro Hemolysis Induced by Streptolysin O and Lysenin

**DOI:** 10.3390/membranes11050364

**Published:** 2021-05-18

**Authors:** Marcelo Ayllon, Gamid Abatchev, Andrew Bogard, Rosey Whiting, Sarah E. Hobdey, Daniel Fologea

**Affiliations:** 1 Biomolecular Sciences Graduate Program, Boise State University, Boise, ID 83725, USA; marceloayllon@u.boisestate.edu (M.A.); gamidabatchev@boisestate.edu (G.A.); andybogard@u.boisestate.edu (A.B.); roseywhiting@u.boisestate.edu (R.W.); 2 Department of Physics, Boise State University, Boise, ID 83725, USA; 3 Idaho Veterans Research and Education Foundation and the Infectious Diseases Section, Veteran Affairs Medical Center, Boise, ID 83702, USA; Sarah.Hobdey@va.gov

**Keywords:** liposomes, pore-forming toxins, hemolysis, virulence factors, streptolysin O, lysenin

## Abstract

The need for alternatives to antibiotics in the fight against infectious diseases has inspired scientists to focus on antivirulence factors instead of the microorganisms themselves. In this respect, prior work indicates that tiny, enclosed bilayer lipid membranes (liposomes) have the potential to compete with cellular targets for toxin binding, hence preventing their biological attack and aiding with their clearance. The effectiveness of liposomes as decoy targets depends on their availability in the host and how rapidly they are cleared from the circulation. Although liposome PEGylation may improve their circulation time, little is known about how such a modification influences their interactions with antivirulence factors. To fill this gap in knowledge, we investigated regular and long-circulating liposomes for their ability to prevent in vitro red blood cell hemolysis induced by two potent lytic toxins, lysenin and streptolysin O. Our explorations indicate that both regular and long-circulating liposomes are capable of similarly preventing lysis induced by streptolysin O. In contrast, PEGylation reduced the effectiveness against lysenin-induced hemolysis and altered binding dynamics. These results suggest that toxin removal by long-circulating liposomes is feasible, yet dependent on the particular virulence factor under scrutiny.

## 1. Introduction

Secreted pore-forming toxins (PFTs) are a distinct class of molecules that disrupt the barrier function through introducing large, unregulated pores in the host cell membranes [1,2,3,4,5,6]. Bacterial-secreted PFTs are virulence factors that actively participate in the infectious process [7,8,9,10,11]: their biological activity may lead to cell death, creating a microenvironment that promotes pathogen proliferation, weakening the immune response and spreading to other hosts [12,13,14,15]. Interrupting this cascade of events by preventing permeabilization is anticipated to diminish their contribution to infectivity and burden on the immune system, hence promoting clearance and health restoration. 

Similar to many other toxins, PFTs must first interact with the components of cell membranes to exert their biological activity. This interaction is often a multi-step process consisting of binding, oligomerization and the creation of conducting pathways; therefore, interrupting one of these steps may constitute an effective strategy for mitigating the infectivity of cytolysin-releasing pathogens. Molecular inhibitors acting as decoys by mimicking the target and competing for binding have been investigated as effective tools for preventing toxin–membrane interactions [16,17,18,19,20,21]. In the same line, the inhibition of oligomerization or the blockage of the conducting pathway of PFTs constitutes other means for preventing PFT-induced permeabilization [22,23,24].

A more general approach derived from the same inhibitory principle relies on using membrane-based decoys as target binding competitors [25,26,27,28,29,30]. Instead of using individual molecules, this strategy introduces larger membrane-based structures as competitors designed to present a high affinity for target binding. In this endeavor, one promising approach is based on employing liposomes as decoys [25,28,30]. While liposomes do not possess direct antibacterial capabilities, they compete with host cells to bind the toxins, sequester them and contribute to their clearance. Their use for this purpose has several advantages over alternative molecular-based approaches. Liposomes have been investigated for many decades as vehicles for drug-delivery; liposomes loaded with antineoplastic drugs are used for cancer therapy and their pharmacology and side-effects are well documented [31,32,33,34,35,36,37,38]. Their composition may be adjusted to effectively compete with the host cells by including specific molecular targets that present a high affinity for a specific toxin or a class of toxins. For example, experiments conducted with liposomes composed of cholesterol (Chol) and sphingomyelin (SM) proved effective against cholesterol-dependent cytolysins (streptolysin O (SLO), pneumolysin, tetanolysin, α-hemolysin) and phospholipase C [28]. Similar liposomes proved effective for attenuating dermonecrosis induced by Methicillin resistant Staphylococcus aureus by sequestering both the PFT α-hemolysin and the α-type phenol-soluble modulin cytolitic factors [30]. A somewhat similar approach utilized a nanosponge consisting of substrate-supported red blood cell (RBC) bilayer membranes to divert α-hemolysin away from cellular targets [29]. Nanoscale biomimetic particles based on RBC membranes have been proposed for the clearance of pathological antibodies [26], pathogenic bacteria [27] and toxins [27,29]. This body of evidence strongly supports the use of liposomes as effective targets for toxins. 

One of the potential issues for the in vivo application of liposomes as toxin removal tools is the reticulo-endothelial system (RES)/mononuclear phagocyte system (MPS) clearance from circulation [39,40,41,42]. Prior in vivo experiments showed that long-circulating, SM-rich liposomes had a half-maximal decline at 4 h owing to elimination by splenic macrophages, which may lead to the requirement of multiple treatments with liposomes at short time intervals [28]. This may also limit the applicability of liposomes to effectively remove only toxins for which SM is a preferred target. Therefore, a better clinical outcome for a liposome-based treatment may be achieved by improving the circulation time of the liposomes irrespective of their composition. In this respect, PEGylated liposomes (also known as Stealth or sterically stabilized) may present a longer circulation time in the blood stream [40,43]. Although the biodistribution of SM-rich liposomes was considered to be similar to PEGylated liposomes in rabbits [31], size can make a great difference. Only large PEGylated liposomes (i.e., >300 nm diameter) presented a median circulation time of ~4 h, similar to the SM-rich liposomes, while the median half-life of smaller liposomes (i.e., <275 nm diameter) exceeded 20 h [31]. Consequently, one may anticipate that a longer bioavailability may improve the efficacy of the liposome-based treatment of infectious diseases. 

As PEG addition is intended to minimize undesired interactions, we asked whether or not such liposomes are efficient targets for toxins, hence actively contributing to their clearance. To answer this question, we investigated the ability of PEGylated liposomes (herein, long-circulating liposomes) to prevent RBC lysis elicited by two potent pore-forming toxins, SLO [44,45] and lysenin [46,47,48]. Our results suggest that long-circulating liposomes are also effective inhibitors of lysis although their effectiveness for particular toxins may be significantly reduced. In addition, the analysis of the inhibition plots revealed that the toxin binding to target membranes is a cooperative process also influenced by the toxin’s identity and lipid composition of the liposomes. 

## 2. Materials and Methods

### 2.1. Materials

Asolectin (Aso, Sigma-Aldrich, St. Louis, MO, USA), Chol (Sigma-Aldrich), brain SM (Avanti Lipids, Alabaster, AL, USA) and distearoyl-phosphatidylethanolamine-PEG2000 (DSPE-PEG, Avanti Lipids) were purchased either as powders or chloroform solutions and stored at −20 °C. The powders were solubilized in chloroform prior to the liposome preparation. Whole sheep blood in Alsever’s solution (Colorado Serum Company, Denver, CO, USA) was stored at 4 °C and used within several days after receiving it. SLO and lysenin (both from Sigma-Aldrich) were prepared as described below and stored in the freezer until use. All of the buffered solutions were prepared in deionized water (DI H_2_O) using analytical grade chemicals purchased from various vendors. 

### 2.2. Methods

#### 2.2.1. Liposome Preparation and Characterization 

Regular and long-circulating liposomes for capturing lysenin or SLO and preventing RBC hemolysis were prepared using sonication [28] or extrusion [49,50] methods. Regular liposomes capable of binding lysenin were prepared from Aso, Chol and SM. Long-circulating liposomes were produced from the same composition with added DSPE-PEG. To capture SLO, regular liposomes were produced by sonication and extrusion from mixtures of Aso and Chol; long-circulating liposomes comprised an addition of DSPE-PEG. The control liposomes were also prepared without the addition of SM or Chol. The powder lipids were dissolved in chloroform, mixed at specific ratios and dried in a glass vial under vacuum for 24 h. The formed lipid cakes were hydrated for a few hours at 60 °C in phosphate buffer saline (PBS) at pH = 7.4. Following hydration, the mixtures underwent four freezing-thawing cycles, after which the liposomes were prepared by either sonication or extrusion. The sonicated liposomes were prepared with a Misonix S-4000 Sonicator (Misonix, Farmingdale, NY, USA) equipped with a micro-tip immersed in the vials kept on ice during the procedure to avoid overheating the sample. The sonication was performed for ~15 min at 25% amplitude and a power transfer of 6–7 W. Liposome extrusion was performed for a total of 81 passes with an Avanti Lipids extruder (200 nm polycarbonate filters, Avanti Lipids) at 75 °C. After preparation, the liposomes were characterized for an average hydrodynamic diameter and polydispersity index (PDI) by dynamic light scattering (DLS, Malvern ZetaSizer, Malvern Panalytical Inc., Westborough, MA, USA) at room temperature and stored in a refrigerator. For each liposome sample, we analyzed three sets and each set consisted of 13 consecutive runs. Each set provided the corresponding average diameter and PDI, from which we calculated the mean values and standard deviations (*n* = 3) as reported in the main text.

Before the experimentation, the liposome solutions were kept at room temperature for two hours for thermal equilibration. The exact compositions and physical characterization data of the liposomes are detailed in the Results and Discussion section. 

#### 2.2.2. Red Blood Cell Preparation 

The whole sheep blood in Alsever’s solution was centrifuged at 3500 RPM for five minutes and rinsed with PBS. After three centrifugation-rinse cycles, the RBCs were re-suspended in PBS to a density of ~9 × 10^9^ cells/mL (as determined with a bench cytometer). For the hemolysis experiments, the washed RBCs were diluted in PBS as detailed in the Results and Discussion section.

#### 2.2.3. Toxin Preparation

A stock solution of SLO was prepared by dissolving the lyophilized compound (~100 ku) in 0.5 mL freezing buffer (100 mM NaCl, 1 mM EDTA, 50 mM Hepes, 1 mM Tris-(2-Carboxyethyl)phosphine-HCl, 0.02% NaN_3_ and 10% glycerol (pH = 7.2). A stock solution of lysenin was prepared by dissolving 50 µg of the lyophilized product in 0.5 mL DI H_2_O. Further dilutions of the stock solutions were made in PBS. 

#### 2.2.4. Determination of Hemolysis Rate 

The extent of hemolysis for the controls and exposed samples was indirectly determined by both a visual inspection and by measuring the optical absorption presented by the released hemoglobin in the supernatant [47,51,52] with a Varian Carry 5000 spectrophotometer (Agilent Technologies Inc., Santa Clara, CA, USA). The inhibition plots were represented as relative hemolysis (determined from absorption at 576 nm) as a function of the total amounts of added lipids/sample (including the Chol). The relative hemolysis was calculated by using the formula:

(1) $$Hemolysis\%=\frac{A_{s}-A_{c}}{A_{max}}\times 100$$

where *A_s_* was the absorption presented by the sample, *A_c_* was the absorption of the control (an otherwise identical sample but containing no added toxin) and *A_max_* was the absorption recorded for the sample containing the toxin but no liposomes added as inhibitors (this absorbance value was set as 100% hemolysis). The absorption was measured at room temperature with the samples placed in 1 cm path length semi-micro cuvettes. All absorption measurements were performed in triplicate; the absorption spectra were shown as single typical runs and the inhibition curves reported mean values. The preliminary experimentation established that a 20 min exposure of RBC samples to various amounts of toxins at room temperature was sufficient to achieve a steady state of hemolysis. For the data analysis, we adapted the four parameter logistic (Hill slope) equation [53] to describe the relationship between hemolysis and liposome amounts:

(2) $$Hemolysis\%=\frac{1}{1+{\left(\frac{x}{EA_{50}}\right)}^{p}}\times 100$$

where *x* was the amount of lipids (in the liposomes) added to each sample including Chol, *EA*_50_ was the effective amount of lipids that set the inhibition halfway (i.e., 50%) and *p* was the Hill coefficient. 

## 3. Results and Discussions

### 3.1. Liposome Characterization

The detailed compositions and physical characterization of liposomes prepared by sonication or extrusion and utilized in this work are presented in Table 1; liposome characterization by DLS provided the average diameter and polydispersity index (PDI). 

The lipid compositions were adjusted to match the target lipid component in the membrane; for example, SLO requires cholesterol [5,6,45] while binding and pore formation by lysenin is conditioned by the presence of SM in the target membrane [46,48]. In addition, to compare long-circulating and regular liposomes as inhibitors of the lytic activity of the toxins, we prepared them with and without the addition of DSPE-PEG. To evaluate the effectiveness of liposomes with membranes devoid of components considered essential for binding toxins, we prepared two control samples. The control utilized to test that SM was an essential component of liposomes used as targets for lysenin was composed of Aso and Chol (no SM) and a sample containing only Aso was prepared to investigate the ability of cholesterol-free liposomes to clear SLO. As one may observe from the physical characterization data presented in Table 1, relatively uniform liposomes were produced irrespective of the preparation method. The differences between them may be related not only to the utilized method but also to the different lipid compositions. As a general rule, liposomes obtained by extrusion were more uniform than the ones obtained by sonication; however, sonication generally led to smaller liposomes. 

### 3.2. Regular vs. Long-Circulating Liposomes: The Prevention of Lysenin-Induced Hemolysis

**Figure 1:**
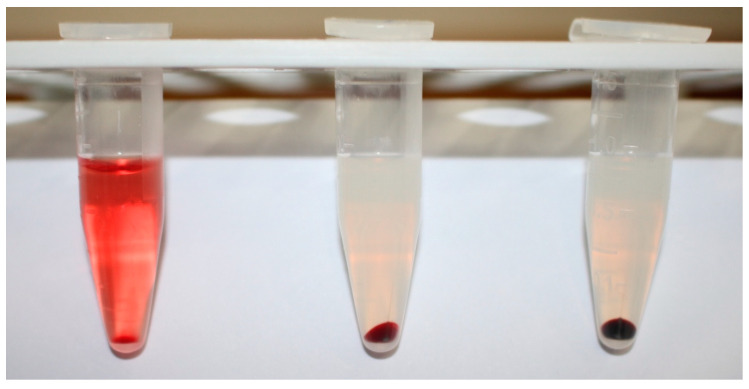
Regular and long-circulating liposomes prevent lysenin-induced hemolysis. The hemolytic activity of lysenin (**left tube**) is canceled by the prior addition of regular (**center tube**) or long-circulating (**right tube**) liposomes prepared by sonication.

To verify the effectiveness of regular and long-circulating liposomes (produced by sonication) as hemolysis inhibitors, we performed a preliminary experiment comprising samples containing ~360 × 10^6^ RBCs in PBS and 10 ng lysenin in addition to either regular or long-circulating liposomes. Through a simple visual inspection of the samples after centrifugation we observed that the absence of liposomes (Figure 1, left) elicited a significant hemolysis as inferred from the red color of the supernatant. The addition of regular liposomes, R1 (~3.5 mg lipids), to the RBC solution prior to the lysenin addition nearly abolished hemolysis by a visual inspection (as indicated in earlier reports) [47,51], rendering the supernatant colorless (Figure 1, middle tube). The addition of an equal amount of long-circulating liposomes, S1, to the RBC solution before the lysenin addition showed a similar result (Figure 1, right tube), i.e., protection against hemolysis. Therefore, we concluded that both regular and long-circulating liposomes may protect the RBCs against hemolysis by providing alternative targets for lysenin binding.

### 3.3. Optimization of Experimental Conditions: The Adjustment of Toxin Amounts

**Figure 2:**
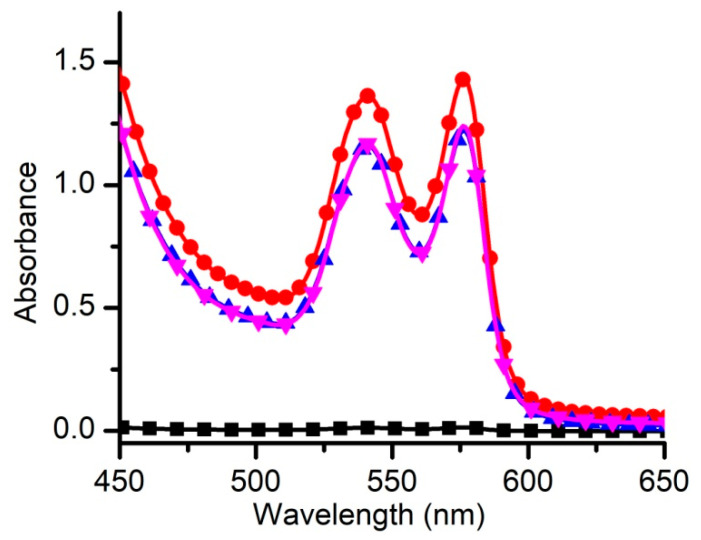
Spectroscopic comparison of Streptolysin O (SLO) and lysenin hemolytic activities. The concentrations of SLO (up triangles) and lysenin (down triangles) were adjusted to yield ~85% of the hemolysis achieved by a strong hypo-osmotic shock (circles). A non-exposed sample (squares) was used as a negative control. Each spectrum represents a typical run; all of the data points are experimental values with the symbols added for easy identification.

The above experiment showed that liposomes may efficiently prevent RBC hemolysis by providing alternative targets for lysenin and clearing the free toxin from solutions. However, our aims included investigating the dependency of inhibition on the concentration and comparing liposome effectiveness as hemolysis protectors for two potent pore-forming toxins, i.e., lysenin and SLO. While the first experiment (see Figure 1) was conclusive with regard to protection against hemolysis provided by both regular and long-circulating liposomes, it was carried with a single, high dose. Both toxins utilized for our investigations have a strong lytic activity stemming from the large conductance pores introduced into the target membranes. However, their mechanisms of primary interaction with membranes are different; their lytic activity depends on the target membrane and solution compositions as well as other environmental factors. The hemolytic activity of the two toxins was not well defined for our specific experimental conditions, which may have introduced difficulties for estimating their lytic efficacy and comparing the effectiveness of liposomes used to mitigate lysis. To overcome these inadequacies, we optimized the amounts of each toxin to achieve similar lytic activities and also hemolysis levels slightly under completion; adding too much toxin to the RBCs may have required very large amounts of liposomes for lysis prevention. For a full hemolysis, we prepared positive controls consisting of 40 µL stock RBCs (~360 × 10^6^ cells) and 360 µL DI H_2_O to produce a strong hypo-osmotic shock. After 20 min, the mixture was centrifuged and a 1:5 dilution of the supernatant into PBS was analyzed by spectrophotometry. After the titration of SLO or lysenin into the RBC solution described above, we found that ~200 units of SLO or ~12 ng lysenin added to the RBC solutions elicited ~85% of the maximum recorded lysis for the water-induced osmotic shock indicative of similar lytic activities (Figure 2).

Throughout the investigations of toxin activities spanning several days, we concluded that SLO’s lytic activity may decrease quite significantly over time. We first suspected RBC degradation as the origin of this behavior. However, over the same time period, lysenin proved very stable with regard to its lytic activity. No sustained lysis was observed for RBCs preserved in the original Alsever’s solution in the refrigerator for up to two weeks. This observation suggests that SLO in solution may undergo a reduction in activity (owing to its oxygen lability) in spite of its preservation in a reductive environment. Therefore, repeating the experiments even over relatively short time periods (i.e., a few days) required the adjustment of the SLO amount added to the RBC solutions so that the lytic activity manifested in the absence of inhibitor liposomes was maintained at ~85% of the full lysis achieved by the hypo-osmotic shock.

### 3.4. Liposomes Inhibit Lysenin-Induced RBC Hemolysis in a Concentration-Dependent Manner

**Figure 3:**
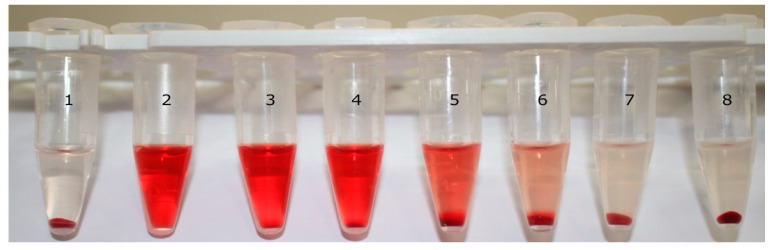
Hemolysis abrogation by increasing amounts of regular liposomes produced by sonication. The non-lysed control sample (1) (red blood cells (RBCs) in PBS) showed an absence of color in the supernatant in contrast to the water-lysed RBC sample (2). Near complete hemolysis was observed for RBCs exposed to lysenin alone (3). Increasing amounts of R1 liposomes (14, 56, 112, 420 and 1680 µg) added to lysenin-exposed samples (4–8) gradually inhibited hemolysis.

We then focused our investigations on exploring the ability of regular and long-circulating liposomes produced by either sonication or extrusion to prevent hemolysis of RBCs exposed to lysenin. Throughout these experiments we used 40 µL of stock RBC solution for a total volume of 400 µL, which included all of the reaction components. For a preliminary visual comparison, we produced samples exposed to ~12 ng lysenin and increasing amounts of sonicated liposomes (R1) together with control samples consisting of RBCs in PBS (no lysenin), RBCs fully lysed by exposure to pure water and RBCs in PBS with lysenin but no liposomes (Figure 3). The qualitative, visual inspection of the reaction tubes indicated a negligible lysis of the control samples (RBCs in PBS) and a full lysis of the samples that comprised a water addition. Strong hemolysis (although not complete) was observed for sample 3, which comprised of a lysenin addition and no liposomes. The clear diminishing of the red color of the supernatants with increasing amounts of liposomes suggested a concentration-dependent inhibition of hemolysis ranging from negligible to full protection.

**Figure 4:**
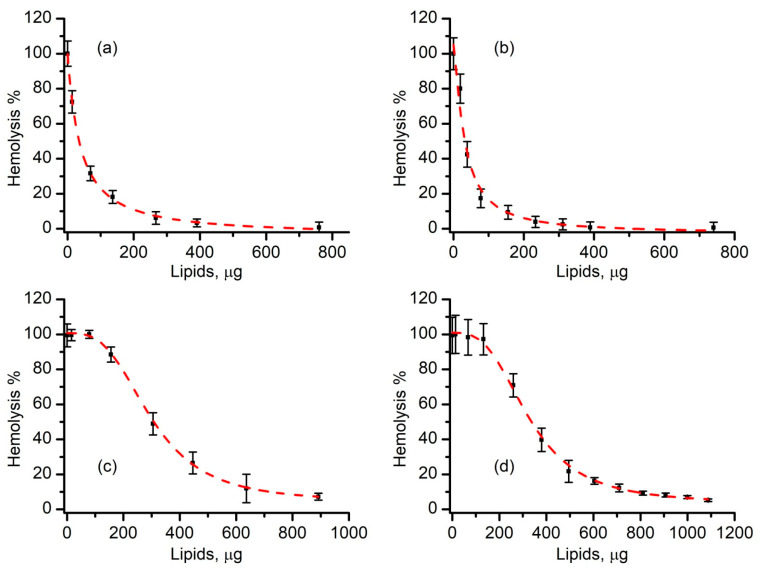
Regular and long-circulating liposomes inhibit lysenin-induced hemolysis in a concentration-dependent manner. Regular sonicated (R1, (**a**)) and extruded (R2, (**b**)) liposomes present similar EA_50_ and cooperativity coefficients. Long-circulating sonicated (S1 (**c**)) and extruded (S2, (**d**)) liposomes present similar EA_50_ and cooperativity coefficients, yet larger than the values determined for regular liposomes. The experimental data (symbols) are reported as mean values (±SD, *n* = 3); the fit with the Hill equation is shown by the dashed line.

The relative hemolysis plots constructed for regular liposomes produced by either sonication (R1) or extrusion (R2) provided additional information on the effectiveness of liposomes as inhibitors of lysenin-induced hemolysis. For both cases, a steep decrease of hemolysis was determined as the amount of added liposome increased. The plots (Figure 4) resembled typical inhibition experiments and they were fitted with the logistic function (Hill) to compare the effectiveness and assess potential cooperative effects. The 50% effective amount (EA_50_) determined for the sonicated liposomes (R1) was 38 ± 3 µg; for extruded liposomes (R2) this amount was 32 ± 5 µg. The Hill coefficient (*p*) was also similar for the two, i.e., 1.2 ± 0.2 for sonicated liposomes and 1.3 ± 0.3 for extruded liposomes, indicative of a slight positive cooperativity.

The ability of long-circulating liposomes produced by either sonication (S1) or extrusion (S2) to prevent lysenin-induced hemolysis was assessed in a similar experimental setup. Both products effectively inhibited hemolysis to negligible values upon the addition of amounts exceeding 200 µg of lipids (Figure 4). A data analysis unveiled more intricate inhibition plots presenting a pronounced sigmoidal shape. In contrast to regular liposomes, which indicated at most a weak positive cooperativity, the Hill coefficient determined for long-circulating liposomes was *p* = 3.1 ± 0.2 for sonicated liposomes and *p* = 3.0 ± 0.1 for the extrusion methods. This suggests cooperativity stronger than what we determined for regular liposomes interacting with lysenin. The corresponding EA_50_s were also larger (298 ± 10 µg for sonicated liposomes and 328 ± 11 µg for extruded liposomes), which were up to 10 times higher than the doses determined for regular liposomes. A reasonable explanation for this reduced effectiveness is that PEGylation modulates the interactions between lysenin and membranes, ultimately influencing binding, oligomerization or pore formation. Such a modulation was expected because liposome PEGylation is intended to prolong the circulation by preventing undesired interactions and the eventual recognition by the immune system of the host but it is not clear why this effect was not observed for SLO. We suspect that the differences between the pore formation mechanisms of the two toxins may play a major role in this modulation.

**Figure 5:**
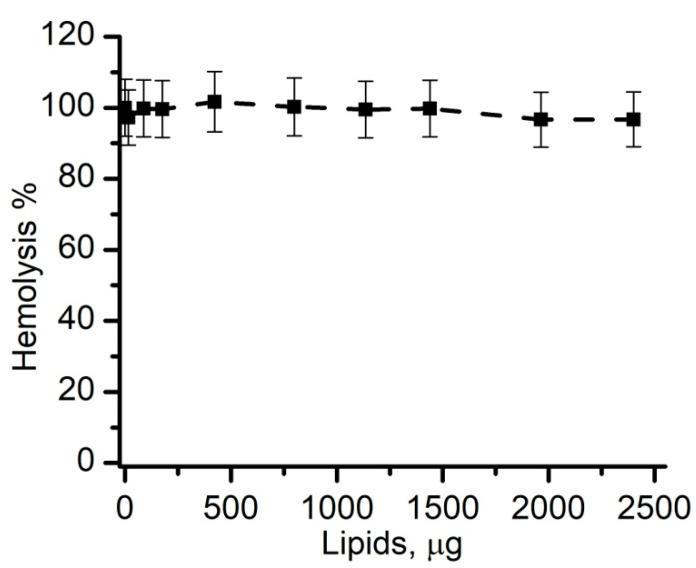
Sphingomyelin (SM) is a required component of the decoy membranes. Lysenin-induced hemolysis was not prevented even by large amounts of extruded liposomes produced without SM (RC1). The experimental data (symbols) are reported as mean values (±SD, *n* = 3). The interrupted line was added as a visual aid.

A large body of evidence indicates the interaction of lysenin with membranes is strongly influenced by the presence of SM into the target bilayer [46,47,48,51,54,55], which is an essential component of the mechanisms leading to binding, oligomerization and pore formation. To further test this requirement, we investigated the inhibitory effects of regular liposomes prepared by extrusion and containing no SM (sample RC1, a replica of sample R4). As expected, no significant hemolysis inhibition was recorded for the addition of liposomes up to 2.4 mg (Figure 5), which was more than double the amount utilized to prevent lysis by similar SM-containing liposomes. Therefore, we concluded that SM was an essential lipid compound for the effective removal of lysenin and the prevention of RBC hemolysis. 

### 3.5. SLO-Induced Lysis Is Prevented by Liposomes in a Concentration-Dependent Manner

**Figure 6:**
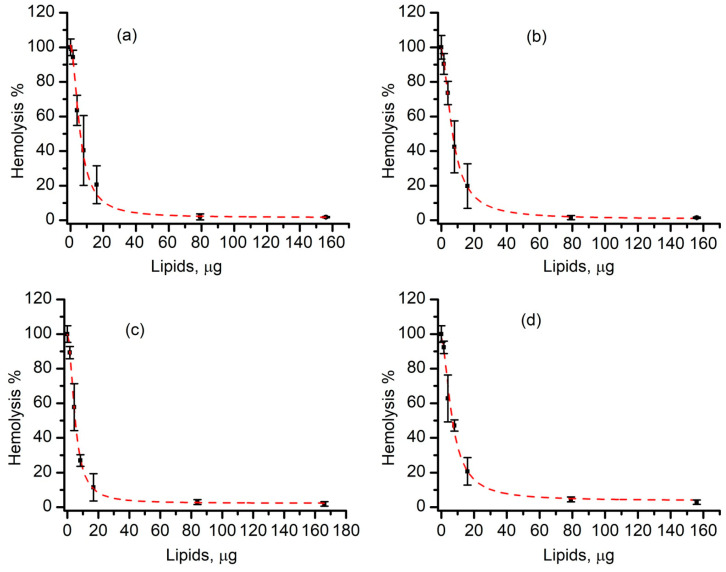
Regular and long-circulating liposomes inhibit SLO-induced hemolysis in a concentration-dependent manner. Regular sonicated (R3, (**a**)) and extruded (R4, (**b**)) liposomes present similar EA50 and cooperativity coefficients. Similar inhibitory patterns are determined for long-circulating sonicated (S3, (**c**)) and extruded (S3, (**d**)) liposomes. The experimental data are reported as mean values (±SD, *n* = 3); the fit with the Hill equation is shown by the dashed lines.

Our next investigations assessed the inhibitory effects of liposomes on SLO-induced hemolysis. The hemolysis rate of RBCs exposed to SLO in the presence of varying concentrations of regular liposomes produced by either sonication (R3) or extrusion (R4) is shown in Figure 6. Irrespective of the production method, the RBC lysis was prevented in an anticipated concentration-dependent manner. The reduction in hemolysis was observed for small amounts of added liposomes and a very steep decrease was observed as the amount of added liposomes increased. Hemolysis was inhibited for lipid amounts exceeding 60 µg, indicative of a near complete prevention of the activity of the toxin. The Hill slope equation provided a similar EA_50_ for both sonicated and extruded liposomes, i.e., 5.8 ± 0.6 µg and 7.0 ± 0.3 µg, respectively. A similar Hill coefficient was also obtained from the fit for both preparation methods (*p* = 1.8 ± 0.1 for sonicated liposomes and *p* = 1.7 ± 0.1 for extruded liposomes) indicating a positive cooperativity, which probably originated in the specific interaction between the toxin and membranes leading to pore formation. All of these results pointed out that sonication and extrusion yielded liposomes with a similar inhibition efficacy of the SLO’s lytic activity. 

The addition of long-circulating liposomes produced by either sonication (S3) or extrusion (S4) also influenced SLO-induced hemolysis and diminished it in a concentration-dependent manner (Figure 6) similar to the regular liposomes. Hemolysis decayed steeply with the amount of added liposomes and the EA_50_ values (5.0 ± 0.6 µg lipids for sonicated liposomes and 6.8 ± 0.9 µg lipids for extruded liposomes) were similar to those determined for regular liposomes as opposed to those observed for lysenin. The Hill coefficients (*p* = 1.8 ± 0.1 for sonicated liposomes and *p* = 1.9 ± 0.2 for extruded liposomes) were also similar to those determined for regular liposomes and were indicative of a slight positive cooperativity and near complete inhibition of hemolysis for amounts exceeding 60 µg of lipids. These results suggested that regular and long-circulating liposomes had a similar efficacy in preventing the lytic activity of SLO irrespective of the production method.

Lysenin and SLO are similar with regard to presenting strong lytic activities that may be diminished by either regular or long-circulating liposome addition. However, a quantitative comparison of the two toxins indicated a few notable differences. For both production methods, the amount of liposomes needed to prevent hemolysis were several times larger for lysenin. The effective prevention of lysenin’s lytic activity (i.e., almost complete abrogation of hemolysis) occurred at lipid amounts exceeding ~10 times the amounts required to prevent SLO-induced lysis. As we adjusted the SLO and lysenin amounts added to the reaction tubes such that they presented similar lytic activities (i.e., 85% of full hemolysis elicited by a strong hypo-osmotic shock), these differences suggested that either lysenin might present a greater lytic activity (which was expected because different mechanisms of interaction with the target membranes were in effect) or that the particular membrane composition utilized for this experiment presented a lower inhibition efficacy. For example, lysenin has been reported to preferentially oligomerize into lipid rafts [55,56,57]; therefore, only a reduced surface area of the liposome is available for binding and pore formation. In contrast, if we assumed that the cholesterol was more uniformly distributed into the membrane of the liposomes produced to prevent SLO-induced lysis, a larger surface area would be available for interaction and the liposomes might remove the SLO toxin in a more effective manner.

### 3.6. Cholesterol-Free Liposomes Do Not Prevent SLO-Induced Hemolysis

**Figure 7:**
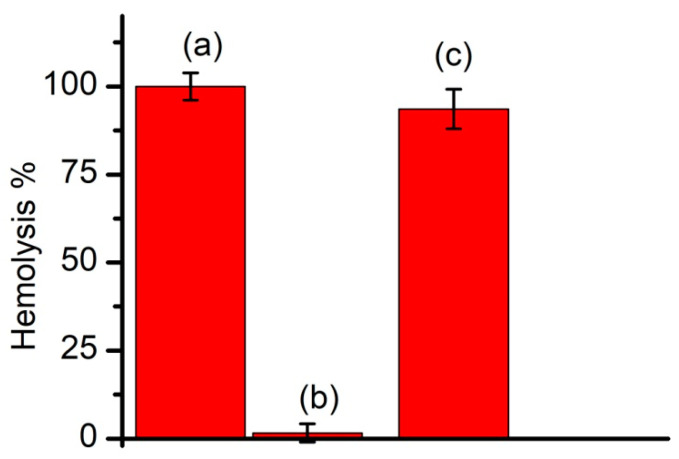
Cholesterol (Chol) is an essential component for the inhibition of SLO-induced hemolysis. A 100% relative hemolysis was recorded for RBCs exposed to SLO only (**a**). The addition of Chol liposomes (R3) abolished hemolysis (**b**). In contrast, the addition of Chol-free liposomes (RC2) presented no inhibitory effects (**c**). The liposomes were produced by sonication and data are represented as a mean ± SD (*n* = 3).

SLO belongs to the larger class of cholesterol-dependent cytolysins; therefore, binding, oligomerization and pore formation are influenced by the presence of cholesterol in the target membranes [58,59]. To assess how the presence of cholesterol in the membranes of liposomes influenced SLO-induced hemolysis, we used Aso liposomes prepared by sonication with and without the addition of Chol to the lipid mixture. The hemolysis experiments were performed by using 1 mg total lipids/RBC sample. SLO alone induced a strong hemolysis of RBCs (Figure 7). However, the addition of an equal amount of liposomes composed of Aso and Chol (sample R3) abolished hemolysis and indicated a complete inhibition. In contrast, the addition of an equal amount of liposomes composed exclusively of Aso (sample RC2) presented a negligible inhibition of hemolysis, confirming that cholesterol was required in the target membrane for strong binding. 

## 4. Conclusions

Our investigations conducted on RBCs indicated that both regular and long-circulating liposomes prepared by sonication or extrusion inhibited the lysis induced by SLO and lysenin. Although liposomes have been tested as tools for toxin removal in vivo [28,30], all of our experiments were conducted in vitro and utilized RBCs as model cells. The use of RBCs as in vitro model systems presents the advantage of enabling a simple and rapid analysis of the experimental data with regard to preventing cells exposed to pore-forming toxins to undergo lysis. In addition, other cells may possess membrane-repair mechanisms [60,61,62] therefore masking the true clearance capabilities presented by liposomes. By investigating this particular biological system in vitro, we were able to test toxin concentrations exceeding what would be typical to in vivo situations. Our results showed that PEGylation and lipid compositions may significantly influence PFT inhibition. Consequently, the adjustment of compositions and concentrations of liposomes may be needed for optimal anti-PFT activity. However, this fact might be partially compensated by the extended availability in the circulation of PEGylated liposomes and also be mitigated by the ability to tailor liposome compositions to specific PFT families (i.e., cholesterol-dependent cytolysins). Finally, as PEGylation and liposome composition may elicit immunological responses that are dependent on the host, load and concentration [63,64], these aspects should be considered for further in vivo investigations on PEGylated liposomes used for PFT clearance. 

## Figures and Tables

**Table 1:** Liposome characterization.

Code *	Composition ** (mg/mL) Aso:Chol:SM:DSPE-PEG	Average Diameter, nm (Mean ± SD, *n* = 3)	PDI (Mean ± SD, *n* = 3)	Preparation Method
R1	5:4:6:0	102 ± 9	0.31 ± 0.06	Sonication
S1	5:4:6:2	95 ± 8	0.28 ± 0.05	Sonication
R2	5:4:6:0	193 ± 3	0.11 ± 0.003	Extrusion
S2	5:4:6:2	214 ± 4	0.09 ± 0.002	Extrusion
R3	10:6:0:0	138 ± 6	0.32 ± 0.07	Sonication
S3	10:6:0:3	144 ± 6	0.22 ± 0.07	Sonication
R4	10:6:0:0	233 ± 11	0.12 ± 0.002	Extrusion
S4	10:6:0:3	238 ± 12	0.09 ± 0.002	Extrusion
RC1	10:6:0:0	236 ± 11	0.11 ± 0.002	Extrusion
RC2	10:0:0:0	268 ± 12	0.33 ± 0.08	Sonication

* R indicates regular (no PEG lipids) and S indicates long-circulating (PEG lipids added) liposomes. ** asolectin (Aso), cholesterol (Chol), sphingomyelin (SM), distearoyl-phosphatidylethanolamine-PEG2000 (DSPE-PEG).

## Data Availability

Not applicable.
